# Supplementary material for: Psychometric Characteristics of Smartphone-Based Gait Analyses in Chronic Health Conditions: A Systematic Review
Source: J Funct Morphol Kinesiol. 2025 Apr 16;10(2):133. doi: 10.3390/jfmk10020133 (PMC12015829; doi:10.3390/jfmk10020133)
Supplement: Supplementary file 1 [file jfmk-10-00133-s001.zip › jfmk-3423841-supplementary.pdf]

Table S1. Characteristics of the included studies.

Reference	Participants			Study design	Intended users / Test location	Disease	Mobilephone (App)	Placement	Gait parameters	Reference (system)
	n	Sex	Age							
Abujrida et al., (*67*)	152	m(NR)/f(NR)	NR	Cross-sectional study	PD-patients / at home	Parkinson	IPhone (mPower)	Pocket	Sway area, gait velocity, cadence, step time, step length, step count	NR
Adams et al., (*40*)	82	m(46)/f(36)	63.3 ± 9.4	Observational study	Parkinson and HC / in clinic & at home	Parkinson	Iphone 10 and Iphone 11 (BrainBaseline™ App)	Hip	Gait speed, step length, stride length	
Alexander et al., (*41*)	100	m(30)/f(70)	Median: 53.5 (IQR: 47.8 - 58.0)	Pilot-/Validationstudy	MS-patients / Clinical (Indoor) & Home (Outdoor)	Multiple Sclerosis	Iphone 6s (mSteps App)	Arm	Distance walked	Trundle wheel
Arora et al., (*69*)	10	m(7)/f(3)	65.1 ± 9.80	Prospektive cohort study	PD-patients / at home	Parkinson	LG Optimus S (Specialized Software)	Hip	NR	Modified UPDRS
Arora et al., (*68*)	334	m(210)/f(124)	66.1 ± 9.0	Prospektive cohort study	PD-patients / at home	Parkinson	LG Optimus S (NR)	NR	NR	Modified UPDRS
Balto et al., (*42*)	45	m(NR)/f(NR)	46.7 ± 10.0	Cross-sectional study	MS-patients / laboratory	Multiple Sclerosis	Iphone 5 / Health (Apple), Health Mate (Withings), and Moves (ProtoGeo Oy)	Pocket	Acceleration, velocity	Digi-Walker SW-200 pedometer (Yamax), UP2 and UP Move (Jawbone), Flex and One (Fitbit)
Banky et al., (*90*)	35	m(22)/f(13)	51.2 (19-85)	Observational, criterion-standard comparison study	Neurological patients / rehabilitation center	Neurological conditions	Samsung Galaxy S5 (NR)	NR	Joint angular velocity	Optitrack 3-D motion analysis
Bourke et al., (*70*)	51	m(24)/f(27)	39.5 ± 7.9	Cross-sectional study	MS-patients / at home	Multiple Sclerosis	Samsung Galaxy S7 (FLOODLIGHT)	Waist in belt bag or pocket	Spatiotemporal parameters	NR

Brinkløv et al., (*91*)	27	m(9)/f(18)	64.2 ± 5.9	Validation Study	Type 2 diabetes patients / field	Type 2 diabetes	Iphone 5c (InterWalk)	Pocket	VO2peak estimation, acceleration vector magnitude	Cosmed K4b^2
Brooks et al., (*43*)	38	m(11)/f(27)	25 - 76	Validation study	CHF- and pHTN-patients / clinic and home	CHF and pHTN	Iphone 4s (SA-6MWT App)	Pocket or Hip holster	Walking distance, step count	ActiGraph accelerometer
Capecci et al., (*44*)	20	m(15)/f(5)	67.6 ± 9.1	Controlled Cross-sectional study	PD-patients / in clinic	Parkinson	Iphone 5 (Specialized Software)	Hip joint	Cadence, freezing index, energy index	Videoanalyse
Chan et al., (*89*)	20	m(11)/f(9)	20 - 65	Observational study	Chronic Low back Pain patients / in clinic	LBP	Iphone 4 (NR)	Lower back	Cadence, step length, velocity, stride time	Minimod
Chen et al., (*71*)	37	m(-)/f(-)	NR	Prospektive cohort study	PD-patients / at home	Parkinson	LG Optimus S (NR)	Pocket	Gait variability	MDS-UPDRS
Cheng et al., (*85*)	76	m(36)/f(40)	39.5 ± 7.9	Cross-sectional study	Clinicans and MS-patients	Multiple Sclerosis	Samsung Galaxy S7	Waist or pocket	Timed 25 Foot Walk	Stopwatch in clinical setting
Chien et al., (*46*)	20	m(2)/f(19)	67.95 ± 7.30	Observational study	Patients with orthostatic tremor / in clinic	Orthostatic tremor	iPhone 6s (custom app)	Sacrum	Mean frequency of acceleration, walking speed	NR
Clavijo-Buendía et al., (*47*)	30	m(15)/f(15)	71.7 ± 5.1	Observational study	PD-patients / in clinic	Parkinson	Samsung Galaxy S8 (RUNZI ®App)	Front Thigh	Cadence, step length, step count, gait velocity	NR
Costa et al., (*48*)	55	m(30)/f(25)	62.5 ± 14.9	Observational study	Stroke survivors / laboratory	Stroke	Iphone 6s and S480 Positivo (Google Fit, STEPZ, Pacer)	Paretic/non-paretic hip pocket	Step count	Actual steps (live and video analysis)
Creagh et al., (*73*)	73	m(23)/f(50)	Mild 39.3 ± 8.3 / moderate 40.5 ± 6.9	Observational study	MS-patients / at home	Multiple Sclerosis	Samsung Galaxy S7 (NR)	Anterior waist	Step count	NR
Creagh et al., (*72*)	52 mild MS; 21 moderate MS	m(16)/f(36); m(7)/f(14)	39.3 ± 8.3; 40.4 ± 6.9	Longitudinal study	MS-patients / at home	Multiple Sclerosis	Samsung Galaxy S7 (Floodlight PoC App)	Pocket or Belt Bag	Only adherence of 2MWT over study duration	Expanded Disability Status Scale (EDSS)
Ellis et al., (*49*)	12	m(7)/f(5)	65.0 ± 8.4	Validity study	PD-patients / in clinic	Parkinson	Apple iPod Touch (SmartMOVE)	Torso (Navel)	Step time, step length	Pressure sensor mat (Steplength) Footswitch (Steptime)

Ginis et al., (*74*)	40	m(23)/f(17)	68.6 ± 6.8	Pilot RCT	PD-patientes / at home	Parkinson	Samsung Galaxy S3 Mini (ABF-gait app and CuPiD)	Pocket (training) and handheld (FOG training)	Gait speed, stride length, double support time (single and dual task)	PKMAS instrumented walkway
Goñi et al., (*82*)	610	m(399)/f(211)	60.3 ± 8.94	Cross-sectional study	PD-patients and HC / remote, self-administered	Parkinson	x/(mPower app)	NR	Average acceleration, number of steps, stride intervall, stride variability	NR
Hamy et al., (*87*)	399	m(NR)/f(NR)	NR	Observational study	RA-patients / remote	Rheumatoid arthritis	Iphone (PARADE App)	Pocket	Step length, step time	GAITRite mat
He et al., (*75*)	119	m(72)/f(47)	64.1 ± 7.9	Observational study	Parkinsonpatients / at home	Parkinson	Iphone 4s and newer (NeuroEnhanceNet)	Pocket	NR	NR
Isho et al., (*50*)	24	m(12)/f(12)	71.6 ± 9.7	Cross-sectional study	Older adults / in clinic	Chronic Stroke	Sony Xperia Ray SO-03C (-)	L3	Trunk acceleration while gait (anteropsoterior, mediolateral) interstride variability	NR
Juen et al., (*51*)	28	m(12)/f(16)	50 - 89	Cross-sectional study	Stroke survivors / laboratory	Pulmonary diseases	Samsung Galaxy S5, Ace (MoveSense)	L3	Walking distance, walking speed (6MWT), step count	Actigraph GT3X
Kim et al., (*23*)	15	m(7)/f(8)	NR	Cross-sectional study	PD-patients / at home	Parkinson	Google Nexus 5 (NR)	Waist, pocket, ankle, chest	Freezing index, acceleration signals	Videoanalyse
Lam et al., (*86*)	94	m(26)/f(68)	46.5 ± 10.6	Longitudinal study	MS-patients and Healthy / remote	Multiple Sclerosis	NR / (MS Sherpa App)	NR	Walking distance	EDSS, T25FW
Lipsmeier et al., (*76*)	43	m(35)/f(8)	57.5 ± 8.45	Prospektive cohort study	PD-patients / at home	Parkinson	Samsung Galaxy S3 Mini (Roche PD Mobile App v1)	Pocket or belt pouch	Turn speed, activity ratio, sit-to-stand transitions	MDS-UPDRS
Lopez et al., (*52*)	10	m(7)/f(3)	45 - 65	Cross-sectional study	PD-patientes / gait lab (MOVISYS)	Parkinson	NR/ (Listenmee app)	NR	Walking speed, stride length, cadence, freezing of gait	Vicon Motion System

Mak et al., (*53*)	110	m(109)/f(1)	68.9 ± 5.9	Observational study	Cardiovascular patients, remote and clinical	Cardiovascular disease	Iphone 7 (VascTrac App)	Pocket	Step count	Clinical Measure "Ground Truth"
Maldaner et al., (*54*)	70	m(43)/f(27)	55.9 ± 15.4	Observational study	Lumbar degenerative disc patients / in clinic	Lumbar degenerative disc disease	(6WT App)	NR	Walking distance	6 min walk normdata and Distance Wheel
Marom et al., (*55*)	28	m(17)/f(11)	42.5 ± 15.0	Cross-sectional study	Rehabilitation patients / in clinic	Unilateral lower limb disability	Xiaomi Redmi Note 8 (OneStep App)	Pockets (front)	Cadence, gait speed, stride length, double support, step length, swing/stance phase	C-Mill VR+ treadmill (Motek)
Mehrang et al., (*77*)	616	m(413)/f(203)	60.6 ± 10.1	Cross-sectional study	PD-patients and HC / at home	Parkinson	Iphone 4s or newer (mPower App)	Pocket or bag	Cadence, step length	NR
Omberg et al., (*78*)	1414	m(481)/f(933)	60	Observational study - remote cohort study	PD-patients/ at home	Parkinson	NR / (mPower)	pocket	Average acceleration, jerk	Clinical measures (ObjectivePD substudy)
Pepa et al., (*56*)	18	m(13)/f(5)	69.0 ± 9.7	Cross-sectional study	PD-patients / in clinic	Parkinson	Samsung Galaxy (NR)	Pocket	Step length, step cadence	Videoanalyse
Pepa et al., (*57*)	44	m(NR)/f(NR)	68.02 ± 8.3	Cross-sectional study	PD-patients / in clinic and a home	Parkinson	Iphone 5, Iphone 6s (NR)	Hip	Step length, step cadence, freezing index, power index, energy derivative ratio	Videoanalyse
Polese et al., (*58*)	37	m(28)/f(9)	62 ± 11	Observational study	Strokepatients / in clinic	Stroke	LG Nexus 5 (Google Fit App)	Paretic lower limb pocket	Step count, walked distance	Actual step count by examiner in videotape
Raknim et al., (*83*)	17	m(7)/f(10)	72.0 ± 6.8	Longitudinal study	Older adults without neurological diseases	Parkinson	Android Smartphones Google, HTC, Samsung (NR)	Pocket	Cadence, step length	NR
Regev et al., (*59*)	100	m(33)/f(67)	40.8 ± 12.4	Cross-sectional study	MSpatients / in clinic	Multiple Sclerosis	NR / (Mon4t Clinic™ app)	Sternum	3m/10m TUG time, tandem walk metrics	EDSS, clinical rater
Rozanski et al., (*92*)	25	m(12)/f(13)	63.9 ± 8.4	Retrospective repeated measures	Patients in rehabilitation program / NR	Neurological or musculoskeletal conditions	NR / (OneStep)	Left/right front or back pocket	Cadence, velocity, hip range, base width, step	NR

									and stride lengths, stance and double support times, asymmetries of stance, step length and double support	
Salvi et al., (*88*)	30	m(11)/f(19)	50 ± 16.6	Longitudinal study	PAH-patients / indoor and outdoor	PAH	Android or iPhone (SMWTApp)	NR	Walking distance (6MWT)	Observational by physiologists
Schwab et al., (*79*)	14	m(NR)/f(NR)	NR	Observational study - remote cohort study	PD-patients / remote at home	Parkinson	NR / (mPowerApp)	Pocket	Tremor, rigidity, freezing of gait, linear and angular acceleration	NR
Serra-Ano et al., (*60*)	29	m(NR)/f(NR)	68.9 ± 8.98	Cross-sectional study	PD-patients / in clinic	Parkinson	Xiaomi Redmi 4x (FallSkip®)	Waist	NR	Videoanalyse
Shema-Shiratzky et al., (*61*)	72	m(35)/f(37)	57.2 ± 1.9	Cross-sectional study	Patients with muscosceletal pathology / in clinic	Muscosceletal pathology (Knee, Back, Hip, Ankle)	Samsung Galaxy A51 (OneStep App)	Thigh	Gait speed, cadence, steplength, cycle time, single- und double-limb support, stancephase	The ProtoKinetics Zeno™ Walkway
Su et al., (*84*)	52	m(33)/f(19)	63 ± 10	Cross-sectional study	PD-patients / in clinic	Parkinson	iPhone (NR)	Front pocket	Stride time, stride time variability	Mobility lab system
Sugimoto et al., (*62*)	22	m(8)/f(14)	21.5 ± 2.56	Cross-sectional study	Recurrent Ankle Sprains patients / biomechanics lab	Recurrent Ankle Sprains	Samsung Galaxy Xcover 2 Model GTS7710L (AccWalker)	Thigh	Sagittal-plane thigh angular RoM	3D motion capture (Qualisys)
Tang et al., (*35*)	20	m(11)/f(9)	73.6 ± 9.1	Observational study	PD-patients / in clinic	Parkinson	Sony Xperia XZ F8331 (NR)	L2	Stride time, step time, stance time, swing time, step length, step velocity, freezing of gait	Xsens MTw Awinda

Tao et al., (*63*)	35	m(23)/f(12)	71.133 ± 8.585	Cross-sectional study	HC and CSVD patients / laboratory	Cerebral small vessel disease	Iphone 13 (MobileGait app)	Shank, waist	Cadence, stride time, stance phase, swing phase, stance time, stride length, walking speed	Inertial Measurement Unit (N200, Wheeltec, China)
Van Oirschot et al., (*80*)	25	m(15)/f(10)	40.0 ± 8.0	Cross-sectional study	MS-patients / at home (outdoor)	Multiple Sclerosis	Android/iOS (MS Sherpa App)	Pocket	2 MW distance, walking speed	Distance markers
Wagner et al., (*64*)	30	m(8)/f(22)	61 (50 - 74)	Validation Study	RA-patients / laboratory	Rheumatoid arthritis	Google Pixel 4, Samsung Galaxy A02 (BeSafe-App)	Waist pouch at right front hip	Step count, walking speed, cadence	Manual step count (100 Steps)
Yahalom et al., (*65*)	18	m(10)/f(8)	50.7 ± 8.8	Cross-sectional study	PD-patients / in clinic	Parkinson	Iphone 6 (EncephaLog)	Sternum	Step length, cadence, mediolateral sway	Videoanalyse
Yahalom et al., (*66*)	21 with normal pull test, 23 with unnormal pull test	m(11)/f(10); m(13)/f(10)	67.3 ± 6.8 / 67.8 ± 6.9	Cross-sectional study	PD-patients / in clinic	Parkinson	IPhone (NR)	Waist	Stride length, cadence, variability	NR
Zhai et al., (*81*)	67	m(25)/f(42)	42.9 ± 10.9	Cross-sectional study	MS-patients / at home	Multiple Sclerosis	Samsung Galaxy S4 mini (NR)	NR	Mean vector magnitude, variance of vector magnitude steps/min	ActiGraph

Abbreviations: CHF = Congestive heart failure, CSVD: Cerebral small vessel disease, EDSS: expanded disability status scale, f: female, HC: healthy controls, m: male, MDS – UPDRS: Modified unified Parkinson´s disease rating scale, MS: Multiple Sclerosis, NR: Not reported, PAH: Pulmonary arterial hypertension, PD: Parkinson Diseases, pHTN: Pulmonary hypertension, RA: Rheumatoid arthritis, RoM: Range of Motion, TUG: Timed up and go test, T25FW: Timed 25-foot walk, UPDRS: Unified Parkinson´s disease rating scale, 2MWT: 2 minute walk test, 6MWT: 6 minute walk test.

Table S2. Psychometric characteristics of the smartphone-based gait analysis across the different pathological diseases.

Reference	Reliability	Validity	Sensitivity	Specificity	Feasibility and Limitations	Main Results
Abujrida et al., (*67*)	NP	NP	96.0% for FoG detection	98.0% for FoG detection	Yes, but noise in home environment affects data	Machine learning accurately classified PD gait impairments, achieving high accuracy (up to 98%) and AUC values (up to 0.99)
Adams et al., (*40*)	Test –retest (ICC > 0.7) Inter/intra-rater reliability NP	NP	NP	NP	Yes	Significant decrease in gait parameters over 12 months
Alexander et al., (*41*)	NP	Yes Content Criterion (concurrent) (95% LOA with-in ± 5 m)	NP	NP	Yes, but GPS accuracy and signal only outdoor	Outdoor GPS from mSteps showed acceptable agreement with the trundle wheel for the MS cohort. Indoor measurements showed high variability.
Arora et al., (*69*)	NP	NP	96.2% for PD discrimination	96.9% for PD discrimination	Yes	Demonstrated excellent discrimination between PD and HC using gait metrics.
Arora et al., (*68*)	NP	NP	91.9% for PD vs control	90.1% for PD vs control	Yes	Smartphones distinguished PD and controls with high accuracy; gait and balance were effective markers.
Balto et al., (*42*)	NP	No significant correlation was found between smartphone applications (Health, Health Mate, Moves) and walking speed ($p > 0.05$) *(12)*	NP	NP	Yes	Smartphone applications lacked the required accuracy and precision for step measurement, making them unsuitable for use in clinical research settings. *(12)*

Banky et al., (*90*)	Test-retest (ICC Absolute = 0.21 - 0.93; ICC Relative: 0.40 - 0.99) Inter/intra-rater reliability NP	Yes Content Criterion (concurrent) (Spearman r ≥ 0.80 for 74.8% of parameters)	NP	NP	Yes, but limited with knee data	Smartphone application showed excellent validity (ICC > 0.8) for velocity, but poor accuracy for the knee.
Bourke et al., (*70*)	Test-retest (ICC = 0.68 - 0.95 for temporal gait parameters; ICC = 0.53 - 0.96 for spatiotemporal, spatial gait parameters) Inter/intra-rater reliability NP	NP	NP	NP	Yes	A single smartphone offers precise and reliable measurements of specific spatial, temporal, and spatiotemporal parameters during a self-administered 2-Minute Walk Test (2MWT). *(12)*
Brinkløv et al., (*91*)	Test-retest (ICC = 0.85 - 0.86) Inter/intra-rater reliability NP	Yes Content Criterion (concurrent) (r2 = 0.45 - 0.60)	98%	77%	Yes, but smartphone placement affects validity; jackets induce higher measurement error	High reliability and validity for VO2-peak prediction with placement in pants. Sensitivity higher than specificity for risk stratification.
Brooks et al., (*43*)	Test-retest (r = 0.94) Inter/intra-rater reliability NP	Yes Content Criterion (concurrent) (r = 0.89; CI = 0.78–0.99)	94%	NP	Yes, but limited to iOS devices	High correlation between app-estimated and in-clinic measured distances (ICC = 0.85 – 0.89). Repeatable at-home results (CoV = 4.6%).
Capecci et al., (*44*)	Test-retest NP Inter/intra-rater reliability (ICC > 0.80)	NP	70.1% (Algorithmus 1) 87.57% (Algorithmus 2)	84.1% (Algorithm 1) 94.97% (Algorithm 2)	Yes, but only in a clinical setting	Algorithm 2 showed significantly higher sensitivity and specificity than Algorithm 1

Chan et al., (*89*)	Test-retest (ICC > 0.4) Inter/intra-rater reliability NP	NP	NP	NP	Yes	The results showed smart phones are feasible for gait tele-monitoring, with potential as prognostic and treatment outcome tools.
Chen et al., (*71*)	NP	Yes Content Criterion (concurrent) (r = 0.54. p < 0.001 for PD severity assessment vs MDS-UPDRS)	97.3% for PD severity discrimination	97.1% for PD severity discrimination	Yes, but requires consistent training and device management	The framework achieved high accuracy and robustness in PD/HC classification and severity estimation
Cheng et al., (*85*)	Test-retest ICC = 0.87 (0.8 - 0.92) Inter/intra-rater reliability NP	Yes Correlation between turn speed at 5UTT and T25FW (r=0.5, p < 0.001)	NP	NP	Yes	A smartphone-based sensor measure for turn speed shows consistent reliability and concurrent validity in evaluating gait and balance impairments in people with Multiple Sclerosis
Chien et al., (*46*)	Test-retest NP Inter/intra-rater reliability (ICC = 0.84–0.92 for mean frequency of acceleration measures (intra-group))	NP	NP	NP	Yes, but limited sample size	Significant mean frequency of acceleration differences between OT patients and controls indicates balance and gait instability in OT.
Clavijo-Buendía et al., (*47*)	Test –retest (ICC = good - excellent) Inter/intra-rater reliability NP	Yes Construct (convergent) (r = 0.424 - 0.957)	NP	NP	Yes	Moderate to excellent correlation with 10-MWT, good to excellent test-retest reliability for RUNZI® parameters

Costa et al., (*48*)	Test-retest<br>(ICC = 0.99 for actual steps;<br>ICC = 0.80 for Pacer iPhone;<br>ICC = 0.68 for Pacer Android;<br>ICC = 0.28 for STEPZ iPhone;<br>ICC = 0.20 for STEPZ Android;<br>ICC = -0.70 for Health iPhone;<br>ICC = 0.10 for Health Android)<br>Inter/intra-rater reliability NP	Yes<br>Content<br>Criterion (concurrent)<br>(r = 0.18 for Health Iphone, p = 0.21)<br>(r = 0.80 for Pacer Iphone, p < 0.01)<br>(r = 0.65 for STEPZ Iphone, p < 0.01)<br>(r = 0.19 for Health Android, p = 0.19)<br>(r = 0.30 for STEPZ Android, p < 0.05)<br>(r = 0.68 for Pacer Android, p < 0.01)	NP	NP	Yes	Pacer (iPhone) showed the highest validity (r = 0.80, p < 0.01) and reliability (ICC = 0.80).
Creagh et al., (*73*)	Test-retest<br>(ICC = 0.91 for Step number;<br>r = 0.47 - 0.52 for T25FW p < 0.01)<br>Inter/intra-rater reliability NP	NP	67.5 % for mild MS<br>80.1 % for moderat MS<br>75.7 for mild vs moderat MS	60.3 % for mild MS<br>87.2 % for moderat MS<br>87.8 for mild vs moderat MS	Yes, but inconsistent device placement significantly impacts measurement accuracy	Models utilizing smartphone features demonstrated superior classification performance, enabling accurate and remote measurements with a single device. These models effectively distinguish gait-related dysfunction in individuals with moderate Multiple Sclerosis from healthy controls and those with mild MS. *(12)*
Creagh et al., (*72*)	NP	Yes<br>Content<br>Criterion (concurrent)	NP	NP	Yes, but e.g., hall length influenced performance	Smartphone-based assessment via DCNN accurately estimated MS-related disability. Severity scores strongly correlated with

		(95% LOA with-in ± 5 m)				EDSS, but variability noted due to testing conditions.
Ellis et al., (*49*)	NP	Yes<br>Content<br>Criterion (concurrent)<br>(ANOVA: increased gait variability in PD-patients with medium to large effect sizes)	NP	NP	Yes, but only in clinical setting	Highlight specific opportunities for smartphone-based gait analysis to serve as an alternative to conventional gait analysis methods (e.g., footswitch systems or sensorembedded walkways)
Ginis et al., (*74*)	Test-retest<br>(η^2 = 0.29. $p < 0.001$)<br>Inter/intra-rater reliability NP	NP	NP	NP	Yes,but device placement unspecified	CuPiD showed greater improvements in gait speed (9%) and dual-task speed (13.5%) compared to controls (5.2%, 5.8%).
Goñi et al., (*82*)	NP	NP	3.69%	99.42%	Yes	Gait metrics provided moderate classification performance between PD and HC.
Hamy et al., (*87*)	Test-retest<br>(ICC = 0.86 - 0.91)<br>Inter/intra-rater reliability NP	Yes<br>Content<br>Criterion (concurrent)<br>(r2 = 0.88, $p < 0.001$)	NP	NP	Yes	Smartphone app demonstrated significant alignment with GAITRite measures (step length, step time).
He et al., (*75*)	NP	Yes<br>Content<br>Construct (discrimnativ)<br>(AUC = 0.883)	FNR = 0.053	NP	Yes	NeuroEnhanceNet achieved the highest AUC (0.883) and lowest FNR (0.053) for early PD detection.

Isho et al., (*50*)	Test –retest (ICC > 0.531 - 0.900) Inter/intra-rater reliability NP	NP	72.7%	84.6%	Yes	Interstride variability of mediolateral acceleration is significantly associated with fall history; AUC = 0.745
Juen et al., (*51*)	NP	Yes Content Criterion (concurrent) (ANOVA: F = 1.114e-4 (S5) and 9.36e-5 (Ace). p < 0.001; no significant differences between MoveSense-App, Actigraph GT3X and „Ground Truth")	NP	NP	Yes	Strong alignment with Actigraph GT3X validated by ANOVA.
Kim et al., (*23*)	NP	NP	Waist: 86%. Pocket: 84%. Ankle: 81%	Waist: 91.7%. Pocket: 92.5%. Ankle: 91.5%	Partial, placement consistency critical, noise from loose attachments	Smartphone-based system detected FOG with high accuracy using acceleration and gyroscope data.
Lam et al., (*86*)	Test –retest (ICC = 0.764; 95% CI (0.651 - 0.845)) Inter/intra-rater reliability NP	Yes Content Criterion (concurrent) (Spearmans rank correlation coefficiant: p = -0.43 to -0.64) Construct (convergent) (Mann–Whitney	Moderate to strong correlation with EDSS and T25FW (ϱ = -0.43 to -0.64)	AUC = 0.482 (95% CI [0.333. 0.632]); insufficient for group-level distinction	Yes, but feasible for self-assessment but dependent on GPS signal quality; adherence decreased over time	Group-level analyses lacked sensitivity to detect clinical changes due to variability, but individual-level curve fitting improved s2MWT reliability, identifying significant changes in walking function.

		U. p < 0.05 for EDSS groups)				
Lipsmeier et al., (*76*)	Test –retest (ICC = 0.80 for Gait) Inter/intra-rater reliability NP	NP	Greater sensitivity than UPDRS	NP	Yes, feasible for home use; adherence of 61% in PD participants; requires patient training	Smartphone-based assessments demonstrated excellent reliability and validity, detecting subtle PD motor impairments and correlating well with MDS-UPDRS ratings. Gait-related impairments were detected using passive monitoring.
Lopez et al., (*52*)	Test –retest (Wilcoxon, p = 0.0117) Inter/intra-rater reliability NP	NP	NP	NP	Yes, but device placement unspecified; limited generalizability due to small sample size	Gait metrics improved significantly with auditory cues: walking speed (+40.6%), cadence (+30.2%), and stride length (+50.3%) compared to baseline.
Mak et al., (*53*)	Test –retest (Cronbach´s Alpha = 0.74) Inter/intra-rater reliability NP	Yes Content Criterion (concurrent) (Cronbach´s Alpha = 0.99)	69%	95%	Yes	High correlation between clinical and remote measurement; reliable for detecting frailty
Maldaner et al., (*54*)	Test –retest (ICC = 0.82; SEM = 58.3 m) Inter/intra-rater reliability NP	Yes Content Construct (convergent) (Pearson correlation coefficients:	NP	NP	Yes, but only outside for GPS	The smartphone app-based measurement of the 6WT is a convenient, reliable, and valid way to determine objective functional impairment in patients with lumbar degenerative disc disease

		moderate r = -0.31 - -0.42)				
Marom et al., (*55*)	Test-retest (ICC = 0.77 - 1) Inter/intra-rater reliability NP	Yes Content Criterion (concurrent) (r = 0.65 - 1)	NP	NP	Yes	The app showed good-to-excellent reliability and moderate-to-excellent validity for all parameters, except step length of impaired leg (poor-to-good).
Mehrang et al., (*77*)	NP	Yes Content Criterion (concurrent) (Random forest classifier, accuracy = 70%)	70%	70%	Yes	Identification of PD via step parameters with 70% accuracy (random forest classifier)
Omberg et al., (*78*)	NP	Yes Content Criterion (concurrent) (r = 0.71, p < 1.8×10^{-6} with UPDRS)	NP	NP	Yes	Strong correlation between gait metrics collected remotely and UPDRS; While remote assessment demands careful interpretation of RWD, our findings support smartphones and wearables for objective, personalized disease evaluation
Pepa et al., (*56*)	NP	NP	85.6% for Algorithm 1 and 2	93.4% for Algorithm 1 and 2	Yes, but requires calibration	Demonstrated high reliability and validity in measuring step length and cadence.
Pepa et al., (*57*)	NP	NP	84.9% for FoG detection	95.2% for FoG detection	Yes, but requires calibration	Demonstrated high validity and sensitivity in freezing of gait detection using fuzzy logic algorithms.

Polese et al., (*58*)	NP	Yes<br>Content<br>Criterion<br>(concurrent)<br>(ICC = 0.93; CI: 0.86 - 0.96)<br>(r = 0.89, p < 0.001)	NP	NP	Yes	Google Fit® application showed excellent agreement (ICC = 0.93) and high correlation with actual step counts.
Raknim et al., (*83*)	NP	NP	94%	NP	Yes, but only on Android	Applying smartphone sensor data to provide early warnings to potential PD patients Classification of changes in gait pattern with 94% accuracy for PD diagnoses
Regev et al., (*59*)	NP	Yes<br>Content<br>Construct<br>(discriminative)<br>(χ^2 test, p < 0.05; AUC = 85.65%)	Sensitivity 75.86% (MS vs. HC. AUC = 85.65%)	Specificity 76.74% (MS vs. HC. AUC = 85.65%)	Yes, but feasible in clinical settings; requires standardized tasks; lacks real-world data	Digital markers differentiated MS patients from HC with AUC = 85.65%; correlations with EDSS (ϱ = 0.55–0.65)
Rozanski et al., (*92*)	NP	Yes<br>Construct<br>(discrimnativ)<br>(g = 0.32 - 0.48)	NP	NP	Yes	Active recordings showed higher stride length, velocity, and lower double support compared to passive recordings.
Salvi et al., (*88*)	Test-retest<br>(ICC = 0.91; SEM = 36.97 m;<br>CoV: 12.45%)<br>Inter/intra-rater reliability NP	Yes<br>Content<br>Criterion<br>(concurrent)<br>(r = 0.89, p < 0.001)	NP	NP	Yes	App measurements strongly correlated with physiologist-observed 6MWD (r = 0.89). ICC for repeatability was 0.91.
Schwab et al., (*79*)	NP	Yes<br>Content<br>Criterion<br>(concurrent)	43%	95%	Yes	Smartphone diagnostics achieved AUC of 0.85 with strong predictive performance for gait-based PD diagnosis.

		(AUC = 0.85; CI: 0.81 - 0.89)				
Serra-Ano et al., (*60*)	Test –retest (ICC = 0.89 - 0.92 for gait) Inter/intra-rater reliability NP	NP	NP	NP	Yes	Reliable differentiation of postural and gait parameters between PD and HC groups.
Shema-Shiratzky et al., (*61*)	Test –retest (ICC = 0.460 - 0.997) Inter/intra-rater reliability NP	Yes Content Criterion (concurrent) (95% LOA)	NP	NP	Partial	High correlation for cadence and gait cycle time (r = 0.996-0.997), moderate correlation for stride length and bipedal support
Su et al., (*84*)	NP	Yes Content Criterion (concurrent) (r = 0.99, p < 0.001 for stride time. r = 0.98 - 0.99, p < 0.001 for stride time variability)	NP	NP	Yes	Demonstrated excellent reliability and validity for stride time and stride time variability in PD patients.
Sugimoto et al., (*62*)	NP	Yes Content Criterion (concurrent) (Group-by-limb interactions for sagittal-plane ankle kinematics F(1,42) = 63,786 p < 0.01; Group-by-limb interactions for	NP	NP	Yes, but requires specific positioning of the device on the thigh	AccWalker effectively detected differences in thigh RoM between RAS and healthy controls.

		sagittal-plane average thigh angular range-of-motion F(1,42) = 6,166 p < 0.017)				
Tang et al., (*35*)	Test-Retest-Reliability (ICC 0.768 - 0.896) Inter/intra-rater reliability NP	Yes Content Criterion (concurrent) (r = 0.858)	90.6%	94.3%	Yes	High consistency between smartphone and XSens; sensitivity and specificity good for FoG detection
Tao et al., (*63*)	Test-retest: (ICC: Thigh = 0.877–0.999; Waist = 0.784–0.996) Inter/intra-rater reliability NP	Yes Content Criterion (concurrent) (Regression analysis: p > 0.05 for most parameters (low/normal speed); no significant differences between the gait parameters of different gait velocitys)	NP	NP	Yes	Reliable for healthy individuals and CSVD patients; higher reliability for thigh placement than waist.
Van Oirschot et al., (*80*)	Test-retest (ICC = 0.649) Inter/intra-rater reliability NP	Yes Content Criterion (concurrent) (ICC = 0.82)	NP	NP	Yes, but GPS accuracy and signal only outdoor	The smartphone-based assessment provided a reliable and valid method for assessing gait speed and distance in persons with MS It enabled remote monitoring and offered a user-friendly solution

						for capturing real-world functional mobility data
Wagner et al., (*64*)	NP	Yes Content Criterion (concurrent) (Spearman's rho for Pixel vs. observed steps = 0.141. p = 0.075); (Spearman's rho for Samsung vs. observed steps = 0.033, p = 0.680)	NP	NP	Yes, but bad results with low walking speed	Spearman correlations between Pixel and observed steps were weak (rho = 0.141), while Samsung showed minimal correlation (rho = 0.033). Accurate at moderate speeds; challenges at low walking speeds
Yahalom et al., (*65*)	NP	Yes Content Criterion (concurrent) (F = 4.4 - 25.4. p < 0.05 for difference between PD and HC	NP	NP	Partial, requires controlled clinical setup, potential impact of psychiatric conditions	Quantitative gait analysis was more sensitive than UPDRS for detecting NIP-related gait impairments.
Yahalom et al., (*66*)	NP	Yes Content Criterion (concurrent) (r = 0.14 - 0.46, p < 0.05 for gait	NP	NP	Yes, but requires smartphone placement training; accuracy depends on	Reliable and valid for measuring stride length and cadence in PD patients under real-world and clinical conditions.

					environmental factors	
Zhai et al., (*81*)	NP	Yes<br>Content<br>Criterion (concurrent)<br>(ρ = 0.29, p = 0.022 for step/min)	57 % for varVM<br>75 % for steps/min	84 % for varVM<br>59 % for steps/min	Yes	Smartphone-based accelerometry offers a more accurate assessment of mobility and disability in individuals with MS compared to wrist-worn accelerometers. Additionally, smartphones effectively differentiate between individuals with MS, healthy controls, and various stages or conditions of MS.

Abbreviations: Ace: Samsung Galaxy Ace, ANOVA: Analysis of variance, AUC: Area under the curve, CI: Confidence interval, CoV: Coefficient of Variation, DCNN: Deep convolutional neural networks, EDSS: Expanded disability status scale, FNR: False negative rate, FoG: freezing of gait, GPS: Global positioning system, HC: Healthy control, ICC: Intraclass correlation coefficient, LoA: Limits of agreement, MDS-UPDRS: Modified Unified Parkinson's Disease Rating Scale, MS: Multiple Sclerosis, NP: Not Provided, OT: Orthostatic tremor, PD: Parkinson disease, p: Statistical significance, r: Pearson correlation coefficient, SEM: Standard error of the mean, S2MWT: Smartphone-based 2 minute walking test, S5: Samsung Galaxy S5, T25FW: Timed 25-foot walk, UPDRS: Unified Parkinson's Disease Rating Scale, varVM: Variance of vector magnitude, VO2: Volume of oxygen, 2MWT: 2 minute walk test, 5UTT: 5 U-Turn test
